# A Meaningless Act, and What Came of It

**Published:** 1885-07

**Authors:** 


### "A MEANINGLESS ACT" AND WHAT CAME OF IT.

Certain Medical Journals, which accused the American Medical Association of doing "a meaningless act" in enlarging the committee of arrangements for the approaching Medical Congress, and instructing them to revise the list of appointments as made by the original committee, must feel very badly to see their charges so thoroughly refuted, as the result of the Chicago meeting shows them to be. These turbulant organs, moreover, have defended the appointment of new code men, and the selection of three hundred and sixty-eight appointees all from the cities of the northeast. They argued that a committee appointed by an organized body immediately acquired powers higher, and superior to, those of the body appointing, and held up for new code men to the last, in spite of right or reason. The bottom was completely knocked out of their position by the opinion of ex-Speaker Randall, given at request of Drs. Gross and Garnett.

This opinion was read at Chicago, and, by resolution of the committee, was ordered to be published in the Association's Journal.

Mr. Randall sustains the action of the Association at New Orleans, and says the committee, as enlarged, superceeds the original committee.

It is very gratifying that the members of the original committee so promptly accepted the decision and the situation, and that two, at least, of the most prominent members of the original seven attended the Chicago meeting, and were in full accord with the committee, all working harmoniously. It is also gratifying to note the committee at Chicago made little alteration in the programme as mapped out by their predecessors, and that with one exception (a notable one, Dr. Robt. Battey, who was really appointed by the first committee) they appointed *not a single one* of their number to any position ; thus giving a striking refutation to the accusations of those Journals that these gentlemen were "sore heads" and "outs," and that their object was to get the "ins ;" and that certain gentlemen "desired to gain prominence in connection with the Medical Congress." The most important and only changes made, except the substitution of the names of gentlemen known to be in affillia- tion with the American Medical Association, for those of new code men wherever they appeared on the list, was the distribution of the offices over a wider area, particularly the chairmen of sections. Instead of five chairmen of sections being appointed from New York, and four from Philadelphia—more than *half* of the entire number of chairmen—the committee at Chicago reduced the number of chairmen in those cities to three each, and gave the others to the following cities, in the following order : Chicago, two ; Cincinnati, O., one ; St. Louis, one ; Louisville, one ; New Orleans, one ; Rome, Ga., one ; Baltimore, one ; Boston, one ; U. S. Army, one.

When the full report of the committee is published it will be seen that a very fair and general distribution of the appointments has been made, and we venture the assertion that the standard of professional qualifications has not thereby been perceptibly lowered.

Following is a list of the presidents of sections as amended: (Those marked * are the changes from the original list.)

SEC. I. *Medical Education, Legislation and Registration:*
PRESIDENT, * Prof. Chaille, of New Orleans, vice Bowditch, of Boston. Dr. Geo. Cupples, of Texas, is First Vice-President of this Section.

SEC. II. Anatomy. President, Prof. Leidy, of Pa.

SEC. III. Physiology. President, Prof. Dalton, of N. Y.

SEC. IV. Pathology. President, Prof. Delafield, of N. Y.

SEC. V. Prac. Med. President, Prof. DaCosta, of Pa.

SEC. VI. Surgery. President, Prof. Yandell, of Ky.

SEC. VII. Obs. and Gynecology. President, Prof. Battey, of Georgia.
(Dr. Hadra, of Texas, is on this Section.)

SEC. VIII. Ophthalmology. President, * Prof. Williams, of Ohio. (Vice Noyes, of N. Y.)

SEC. IX. Otology. President, Prof. Blake, of Boston.

SEC. X. Dermatology and Syphilis. President, Prof. Hardaway, of Mo.
(Dr. F. E. Daniel, of Tex., Sec'y of this Sec.)

SEC. XI. Laryngology. President, *Prof. McKenzie,of Md. (Vice Lefferts, of N. Y.)

Sec. XII. Pub. and Intern'l Hygiene. President, Prof. Johnson, of Ill.
(Dr. Swearingen, of Texas, is on this Sec.)

SEC. XIII. Collective Investigation, Nomenclature and Vital Statistics. President, Prof. N. S. Davis, of Chicago.
(Dr. H. C. Ghent, of Texas,is on this Sec.)

SEC. XIV. Military and Naval Surgery. President, Surgeon Huntington, U. S. A.

SEC. XV. Practical and Experimental Therapeutics. President, Prof. H. C. Wood, of Phila.
(Dr. Paine, of Galveston, is on this Sec.)

SEC. XVI. Diseases of Children. * President, Prof. L. Lewis Smith, of N. Y. (Vice Jacobi, of N. Y.)
(Dr. Pope, of Texas, is on this Sec.)

This is as it should be. The medical profession *of America* is more nearly represented than it was before; and we trust now that those gentlemen who were dissatisfied with the action of the association at New Orleans will abide by the decision of their own chosen umpire, Speaker Randall, who is, since the days of Cushing, recognized as the *highest authority* on Parliamentary Law, and that the work of organization will go on smoothly, and that we will have an International Congress in America, of which no American will be ashamed.

P. S. We had just finished the above when we learned from the two New York medical weeklies that a number of the Philadelphia physicians, twenty-eight out of the four hundred, most of whom held prominent appointments, had split off, and refuse to have anything to do with the Congress. Included in this number we regret to see the name of Dr. D. W. Yandell, of Louisville, President of the Section of Surgery, who was present by invitation. We are surprised to see the name of Dr. S. W. Gross there, also, in as much as *he is the one who submitted the question to Speaker Randall for decision.* He now refuses to abide by the decision! How inconsistent! If the umpire had been of the selection of the opposite party or faction, this action would not have been so surprising. It only shows a spirit of rule or ruin, and clearly fixes the blame for turbulence and discord on that party generally known as 'specialists.'

Here it is, and the reason (?) assigned for it.

The *New York Medical Journal* says:

"After hearing a report of the proceedings of the new committee at the meeting held in Chicago last week, and after considering the changes in the organization which were made *including* [the removal of—ED.] *the restrictions of the scope of the membership, by which a large proportion of the profession of the country would be excluded from the Congress,* [italics ours.—ED.] the following preamble and resolutions were unanimously adopted:

*Whereas*, Certain serious changes have been recently effected in the preliminary organization and rules for the International

Medical Congress of 1887, it has seemed desirable for the members of the General Committee and the officers of the Sections resident in Philadelphia to meet for consultation ; and

*Whereas*, It has appeared that these changes are inconsistent with the original plan, and detrimental to the interests of the medical profession in America, and of the International Medical Congress ; therefore be it

*Resolved*, That we, the undersigned, consider that our duty to the profession and to ourselves requires us to decline to hold any office whatsoever in connection with the said Congress as now proposed to be organized :

| | |
|---|---|
| D. HAYES AGNEW, | S. WEIR MITCHELL, |
| ROBERTS BARTHOLOW, | WILLIAM F. NORRIS, |
| JOHN H. BRINTON, | WILLIAM OSLER, |
| CHARLES H. BURNETT, | JOHN H. PACKARD, |
| R. A. CLEEMANN, | THEOPHILUS PARVIN, |
| J. M. DACOSTA, | WILLIAM PEPPER, |
| LOUIS A. DUHRING, | EDWARD T. REICHART, |
| WILLIAM H. FORD, | ALBERT H. SMITH, |
| WILLIAM GOODELL, | ROBERT MEADE SMITH, |
| SAMUEL W. GROSS, | ALFRED STILLE, |
| ROBERT B. HARRISS, | GEORGE STRAWBRIDGE, |
| I. MINIS HAYS, | WILLIAM THOMSON, |
| WILLIAM W. KEEN, | JAMES TYSON, |
| JOSEPH LEIDY, | HORATIO C. WOOD, |

DAVID W. YANDELL.

It seems these gentlemen are mad because the restriction *was* removed, and persons were appointed from elsewhere in America than Philadelphia, thus reasserting that all the medical talent of America is resident there. They say the action of the new committee "is inconsistent with the original plan." Well, we should say it is ; the "original plan" being to fill all the positions with physicians from Philadelphia and New York, and most of them new code men. Thus, these gentlemen "beg to differ" with the American Medical Association and with the *recognized authority* on Parliamentary Law after having—themselves—submitted the question to his decision. This action will subject those distinguished gentlemen to severe and just criticism. They have acted with a petulence and want of consistency more becoming a school boy who could not be allowed to have his own way in spite of reason and authority ; and we regret to see it, not that the American Medical Association cannot afford to do without them—not that there could be no International Congress without them, but because their example is likely to be followed by other cities, and thus break up the Congress and the American Medical Association as well. We expected those who were thrown out to stay out, but these gentlemen are not new code men; as before said, many of them occupying the highest positions by appointment by the original committee, endorsed by the new committee. We utterly fail to see how the action of the new committee in widening the scope,and taking in a few well-known gentlemen from elsewhere than Philadelphia, can prove "detrimental to the interests of the medical profession of America." That is a mere pretext; *they* are the "sore heads"—they were not permitted to "run" the Congress their own way—despite the American Association, and even after their umpire *had told them they were wrong*! and they would, therefore, in a spirit of "rule or ruin," burst up the whole Congress.

We hope the committee will loose no time in filling their places, which can easily be done; but it is a matter very much to be regretted that these gentlemen should have seen proper to take such action. They might have waited till their appointment had been officially announced,—as it is, they are a little previous ; and as to Prof. Yandell, he did not have a walk over in getting the appointment. He reminds us something of old Dog Tray—caught in bad company.

